# Type III interferons attenuates Th1/Th17 cell pathogenicity and regulates retinal pigment epithelium cells via NLRP1/NLRP3 signaling axis in autoimmune uveitis

**DOI:** 10.1016/j.gendis.2025.101957

**Published:** 2025-11-27

**Authors:** Wujiao Wang, Qingfeng Wang, Jinyu Cai, Guannan Su, Wanyun Zhang, Shuai Su, Yunfan Zheng, Chaokui Wang, Peizeng Yang

**Affiliations:** a Ophthalmology Medical Center, The First Affiliated Hospital of Chongqing Medical University, Chongqing Key Laboratory for the Prevention and Treatment of Major Blinding Eye Diseases, Chongqing Branch (Municipality Division) of National Clinical Research Centre for Ocular Diseases, Chongqing 400016, China; b Department of Urology, Urologic Surgery Center, Xinqiao Hospital, Third Military Medical University (Army Medical University), Chongqing 400037, China; c Department of Ophthalmology, Henan Province Eye Hospital, Henan International Joint Research Laboratory for Ocular Immunology and Retinal Injury Repair, The First Affiliated Hospital of Zhengzhou University, Zhengzhou, Henan 450000, China

**Keywords:** NLRP1, NLRP3, Retinal pigment epithelium, Type III interferon (IFN-λ), Uveitis

## Abstract

Accumulating data implicate Type III interferons (IFN-λs) in autoimmune disorders, prompting our exploration of their role in uveitis pathogenesis. Serum and peripheral blood mononuclear cells (PBMCs) from patients with active Vogt-Koyanagi-Harada (VKH) and active Behçet's disease (BD) were analyzed for IFN-λ expression by enzyme-linked immunosorbent assay and real-time quantitative PCR. Experimental autoimmune uveitis (EAU) was induced in IFNLR1^−/−^ mice to evaluate disease severity, inflammatory responses, and blood-retinal barrier (BRB) integrity. RNA sequencing and bioinformatic analyses were performed to identify related genes and associated signaling pathways. IFN-λ levels were significantly elevated in active VKH and BD patients and effectively distinguished them from healthy controls. Compared with wild-type mice, IFNLR1^−/−^ mice developed more severe EAU, characterized by increased Th1/Th17 responses, reduced Treg frequency, and disrupted blood-retinal barrier integrity, which was evidenced by decreased tight junction proteins ZO-1, Claudin-5, and Occludin. Both retinal pigment epithelium (RPE) cells from IFNLR1^−/−^ mice and human primary retinal pigment epithelium (RPE) cells with silenced IFNLR1 secreted higher levels of interleukin (IL)-6, IL-8, IL-1β, and MCP-1, which were suppressed by recombinant IFN-λ1 and IFN-λ2. RNA sequencing revealed an enrichment of T-cell and NOD-like receptor signaling pathways in IFNLR1^−/−^ EAU mice. Consistent with this transcriptional profile, the expression of NLRP3 and NLRP1 was upregulated in RPE cells. Knockdown of these inflammasomes reduced proinflammatory cytokine production and upregulated the tight junction proteins. These results suggest that IFN-λs may alleviate uveitis by targeting RPE cells, primarily through downregulation of NLRP1/NLRP3 inflammasome activity, thereby attenuating inflammatory responses and preserving BRB integrity.

## Introduction

Uveitis is a vision-threatening ocular inflammatory disease with complex etiology, often affecting multiple systems.^1^ Among its various forms, immune-mediated uveitis, such as Vogt–Koyanagi–Harada (VKH) disease and Behçet's disease (BD), represents a major clinical entity in certain populations.^2^ Accumulating evidence underscores the central role of dysregulated CD4^+^ T-cell responses in its pathogenesis, particularly the imbalance between pathogenic T helper 1 (Th1)/Th17 cells and regulatory T cells (Tregs).^3^ To further investigate the mechanisms underlying immune-mediated uveitis, the mouse model of experimental autoimmune uveitis (EAU) induced by retinal antigens has been widely applied for *in vivo* studies.^4^ EAU is currently the best uveitis animal model and provides a reliable tool for studying the pathogenesis of uveitis such as BD and VKH, and for investigating novel treatment methods. EAU shares many similar clinical and histologic characteristics with human autoimmune uveitis, including serous vitritis, choroiditis, and retinitis.^5^

Autoreactive T cells, primed in the periphery, migrate into ocular tissues by crossing the blood-retinal barrier (BRB), a structure composed of the retinal pigment epithelium (RPE) and retinal vascular endothelium, thereby triggering intraocular inflammation and visual impairment.^6^, ^7^, ^8^, ^9^ Under inflammation or external stimulation, disruption of RPE tight junctions increases BRB permeability.^10^, ^11^, ^12^, ^13^ Moreover, RPE cells secrete various inflammatory cytokines, including interleukin (IL)-6, IL-8, MCP-1, and IL-1β, and contribute to uveitis pathogenesis by activating intraocular immune cells and recruiting leukocytes into the eye.^14^^,^^15^

Interferons are cytokines produced in response to infectious or inflammatory stimuli.^16^ These cytokines have potent antiviral effects and modulate immune cell function.^17^ Type III interferons (IFN-λs) consist of three functional genes in humans (IFN-λ1/IL-29, IFN-λ2/IL-28A, IFN-λ3/IL-28B) and two in mice (IFN-λ2/IL-28A, IFN-λ3/IL-28B).^18^^,^^19^ IFN-λs signal through a unique heterodimeric receptor complex comprising IFN-λ receptor 1 (IFNLR1) and IL-10 receptor.^20^ Unlike other interferon receptors, IFNLR1 expression is restricted primarily to epithelial cells and specific immune cells, such as dendritic cells (DCs) in mice and neutrophils in humans, allowing targeted effects at barrier surfaces.^21^ Recently, IFN-λs have been reported to modify the adaptive immune response and play an essential role in autoimmune disease progression.^22^ For instance, IFN-λs exhibit protective anti-inflammatory effects in arthritis, colitis and thromboinflammation by regulating neutrophil function through a translation-independent signaling pathway.^23^, ^24^, ^25^ IFNLR1^−/−^ mice display increased blood–brain barrier (BBB) permeability after viral infection in the central nervous system (CNS), suggesting a barrier-stabilizing role for IFN-λs.^26^ Conversely, IFN-λs may contribute to systemic lupus erythematosus (SLE) pathogenesis, as elevated IFN-λ levels are observed in SLE patients.^27^ These findings suggest that IFN-λs exert significant pro- or anti-inflammatory effects in the context of pathophysiological conditions. However, the role of IFN-λs in uveitis remains unclear. Herein, we investigated the potential role of IFN-λs in this disease and the mechanisms involved.

## Materials and methods

### Ethics, patient consents and registrations

This study included 15 patients with active Behcet disease (BD), 16 patients with active Vogt-Koyanagi-Harada (VKH) patients and 15 normal controls for the detection of the serum level of IFN-λs. Peripheral blood mononuclear cells (PBMCs) were obtained from 11 active VKH patients, 11 active BD patients and 11 normal controls for the analysis of mRNA expression of IFN-λs. The diagnosis of both diseases adhered to international criteria^28^^,^^29^ and modified criteria previously reported by our group.^30^ All patients were treatment-naïve for immunosuppressants or prednisone for at least 2 weeks prior to sampling. Written informed consent was obtained from all participants. The study procedures were complied with the tenets of the Declaration of Helsinki and were authorized by the Ethics Committee of The First Affiliated Hospital of Chongqing Medical University (approval no. K2023-030).

### Animals

IFNLR1^−/−^ mice on a C57BL/6J background were purchased from Shanghai Model Organisms Center, Inc. Mice were housed under specific pathogen–free conditions in the animal facilities at the Experimental Animal Center of Chongqing Medical University. All protocols involving the mice were approved by the Ethics Committee of the First Affiliated Hospital of Chongqing Medical University (approval no. 2023-0060).

### Induction of EAU

Human IRBP_651-670_ (LAQGAYRTAVDLESLASQLT; 500 mg; Sangon, China) was dissolved in 1 mL of phosphate-buffered saline (PBS) emulsified with an equal volume of complete Freund's adjuvant (CFA) (Sigma–Aldrich, St. Louis, MO) containing 1.0 mg/mL *Mycobacterium tuberculosis* strain H37Ra (Sigma–Aldrich). For the induction of EAU, mice were subcutaneously injected with 500 μg of the configured IRBP_651-670_ peptide after intraperitoneal anesthesia, followed by intraperitoneal injection of 1 μg pertussis toxin (List Biological Laboratories, Campbell, CA, USA). All immunized mice were examined by slit lamp from day 8 post-immunization following EAU induction and randomly assigned to different groups. Clinical and histopathological assessments were performed blindly by two researchers according to established criteria.^31^^,^^32^

### Isolation and culture of the mouse and human primary RPE cells

The wild-type (WT) EAU mice and IFNLR1^−/−^ EAU mice were euthanized by cervical dislocation for eyeball extraction. Donated eyeballs were obtained from the Eye Bank of the First Affiliated Hospital of Chongqing Medical University for corneal transplantation, and the remaining tissue after corneal debridement was used for RPE cell isolation. The tissue handling, transport and storage of human RPE were conducted in accordance with the provisions of the International Code of Conduct for the Use of Human Tissues and approved by the Ethics Committee of the First Affiliated Hospital of Chongqing Medical University. RPE cells were isolated and cultured as previously described.^33^^,^^34^ Briefly, eyeballs from human donors or mice were incised at the corneoscleral limbus, and the anterior segment and vitreous were removed. Eye cups were rinsed in PBS for 10 min to facilitate neural retina separation, followed by a 45-min incubation in 0.25% trypsin–EDTA (Beyotime, China) at 37 °C with 5% CO_2_. Trypsinization was stopped by adding DMEM/F12 medium supplemented with 10% fetal bovine serum (FBS). The interior of the eye cups was flushed 20–30 times with a sterile transfer pipette, and the resulting cell suspension containing RPE cells was transferred into a 15 mL centrifuge tube and centrifuged at 2000 rpm for 15 min at room temperature. The supernatant was discarded and the RPE cells were resuspended in 1 mL complete medium (DMEM/F12 + 10% FBS + 1% Penicillin/Streptomycin) and cultured in T25 cell culture flask at 37 °C with 5% CO_2_ for subsequent experiments.

### Evans blue assay

The vascular permeability of the BRB was evaluated by Evans blue as described previously.^35^ On day 14 post-immunization, mice were injected with 100 μL of 2% (weight/volume) Evans blue (no. MB4680; Dalian Meilum Biotechnology Co., Ltd., China) through the tail vein. After 2 h, mice were euthanized, and eyeballs were enucleated and fixed in 4% paraformaldehyde. After removing the redundant tissues, the retinas were dissected, and washed twice in PBS. Retinal whole mounts were prepared and Evans blue leakage was examined by immunofluorescence microscopy (Leica, Germany).

### Hematoxylin and eosin (H&E) staining

Fixed and dehydrated eyeballs were embedded in paraffin wax, and serial 4–6 μm sections were stained with H&E. EAU severity was graded on a scale of 0–4 as described previously.^36^

### Flow cytometry (FCM)

Anti-mouse IFN-γ-PE-cy7, IL-17A-PE, FOXP3-PE, CD25-PE-cy7 and CD4-APC were purchased from Biolegend, USA. Mouse splenic mononuclear cells were stimulated with ionomycin, PMA and brefeldin A (R&D Systems, USA) for 4 h. The cells were stained with anti-mouse CD4-APC, anti-mouse IFN-γ-PE-cy7, anti-mouse IL-17A-PE, anti-mouse CD25-PE-cy7 and anti-mouse FOXP3-PE. The CD11c microbeads were used to isolate splenic DCs. These DCs were collected and incubated with anti-mouse CD86-APC (BioLegend, USA), anti-mouse CD11c-PE (BioLegend, USA), anti-mouse CD80-APC (BioLegend, USA), anti-mouse CD40-PE (BioLegend, USA) and anti-mouse MHCII-FITC (BioLegend, USA) at 4 °C for 30 min. Data were analyzed using FlowJo software (Tree Star, Ashland, OR, USA).

### Enzyme-linked immunosorbent assay (ELISA)

Serum levels of IFN-λ1, IFN-λ2 and IFN-λ3 in patients with active VKH and active BD were measured using human ELISA kits (Mengbio, China). The concentrations of IL-6, IL-12, TNF-α and IL-1β of mouse DCs were measured using mouse ELISA kits (Mengbio, China). Similarly, the concentrations of IL-6, IL-8, IL-1β, MCP-1, ZO-1, Claudin-5 and Occludin in the culture supernatants of RPE cells were determined using ELISA kits (Mengbio, China).

### Small interfering RNA (siRNA) transfection

Small interfering RNA (siRNA) targeting IFNLR1 (5′-GGCGAGGGAAUCAGAAAUU-3′) was synthesized by ChemShine Biotechnology Inc (Shanghai, China). The siRNA was diluted in 50 μL Opti-MEM to a final concentration of 50 nM. Lipofectamine™ 3000 (1 μL) was diluted in Opti-MEM (50 μL), mixed with the diluted siRNA solution, incubated for 20 min at room temperature, and added to cells in 24-well plates (100 μL/well). Cells were incubated for 18–48 h at 37 °C with 5% CO_2_.

### Induction of apoptosis *in vitro*

Human RPE cells, primed with lipopolysaccharide (LPS, 100 ng/mL for 12 h), were pre-incubated in DMEM/F12 medium for 1 h. The cells transfected with siIFNLR1 were subsequently treated with NLRP3 agonists (nigericin at 6 μM for 2 h; monosodium urate (MSU) at 250 μg/mL for 6 h; or silica at 250 μg/mL for 6 h) or an NLRP1 agonist (L18-MDP at 100 μg/mL for 16 h) to activate the inflammasomes, and the cells were harvested for apoptosis analysis. The 10 × Binding Buffer was diluted ten-fold with sterile deionized water to prepare a 1 × working solution. Following detachment with trypsin (without EDTA) and neutralization with complete culture medium, cells were centrifuged at 500 × *g* for 5 min. The resulting pellet was washed twice with ice-cold PBS and subsequently resuspended in the 1 × Binding Buffer to a final concentration of 1 × 10^6^ cells/mL. Aliquots of 100 μL cell suspension (containing 1 × 10^5^ cells) were stained with 5 μL of Annexin V-FITC and 5 μL of propidium iodide (PI), respectively, and incubated for 15 min at room temperature in the dark. Samples were then subjected to analysis by FCM.

### RNA-sequencing, identification of differentially expressed genes (DEGs) and enrichment analysis

Total RNA from mouse RPE cells and DCs was extracted using Trizol reagent according to the manufacturer's instructions. RNA-seq was performed by Novogene (Beijing, China) after confirming that the total RNA quality met the requirements for library construction and sequencing. Sequencing was performed on the Illumina NovaSeq 6000. High-quality clean data was generated by removing unqualified reads from the raw data prior to further analysis. A padj ≤ 0.05 indicated significant enrichment. Differential expression analysis was performed using the DESeq2 R package (version 1.20.0) with criteria of *P* ≤ 0.05 and |log2 fold change| ≥ 1.5. Enrichment analyses for DEGs in Kyoto Encyclopedia of Genes and Genomes (KEGG) pathways and Gene Ontology (GO) terms were conducted using the ClusterProfiler R package (version 3.8.1). Significance thresholds were defined as normalized enrichment score (NES) > 1, nominal *P* < 0.05, or false discovery rate (FDR) *q*-value < 0.25.

### RNA preparation and real-time quantitative PCR (RT-qPCR)

Total RNA from PBMCs and RPE cells was extracted using TRIzol (Invitrogen, San Diego, CA, USA) and reverse transcribed using Prime Script RT reagent kit (Takara, Kusatsu, Japan) according to the manufacturer's protocol. RT-qPCR was performed on an ABI 7500 Real-Time PCR System (Applied Biosystems, California, USA) using SYBR Green Kit (Takara, Kusatsu, Japan). All primers were synthesized by Sangon (Shanghai, China). Relative Gene expression was normalized to GAPDH and calculated using the 2^−ΔΔCt^ method.

### Western blotting assays

Retinal proteins were lysed in RIPA buffer with protease inhibitors (Beyotime, China). Protein concentration was determined using a bicinchoninic acid assay (BCA) kit (Beyotime, China). Equal amounts of protein were separated by sodium dodecyl sulphate-polyacrylamide gel electrophoresis (SDS-PAGE), and electroblotted onto polyvinylidene difluoride (PVDF, Millipore) membranes. The membranes were blocked with 5% non-fat milk, and incubated with primary antibodies overnight at 4 °C, followed by secondary antibodies for 100 min at room temperature. The bands were visualized using an enhanced chemiluminescence kit. The density of the signals was quantified using ImageJ software and normalized to the internal references.

### Statistical analysis

Data are presented as mean ± SEM and analyzed using GraphPad Prism 9.3. Multiple comparisons were corrected using Bonferroni's method. Mann–Whitney *U* tests and unpaired Student's *t* test (2 tailed) were performed to assess significance between two independent groups. One-way ANOVA test was applied for multiple group comparisons. *P* < 0.05 was considered statistically significant.

## Results

### IFN-λs are increased in both active BD and active VKH

**Figure 1:**
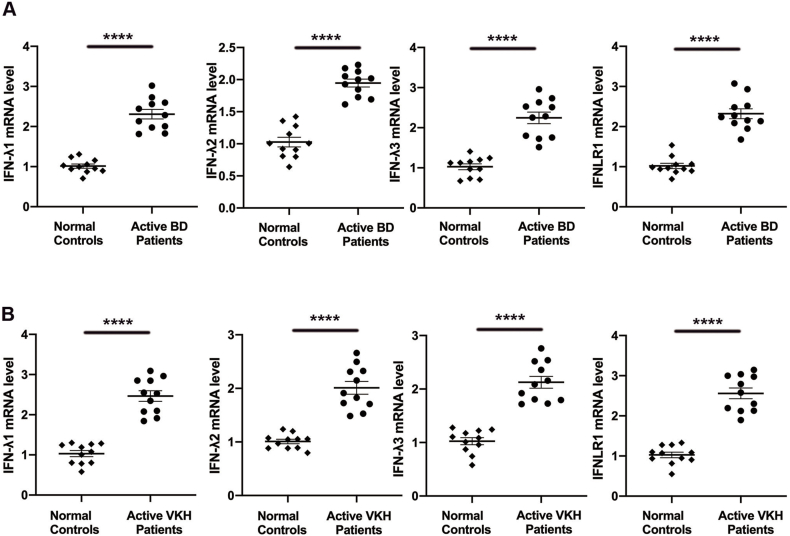
mRNA expression of IFN-λs and IFNLR1 in patients with active BD and active VKH. PBMCs were collected from active VKH, active BD and normal controls, and the mRNA expression of IFN-λs and IFNLR1 was measured by RT-qPCR. **(A)** 11 active BD patients *vs.* 11 normal controls. **(B)** 11 active VKH patients *vs.* 11 normal controls. Data are expressed as mean ± SEM, and dots represent individual participants. Statistical significance was determined using Mann–Whitney *U* tests or independent *t* tests. ∗∗∗∗*P* < 0.0001. BD, Behçet's disease; VKH, Vogt-Koyanagi-Harada; PBMCs, peripheral blood mononuclear cells; IFN-λs, Type III interferons; RT-qPCR, real-time quantitative PCR.

**Figure 2:**
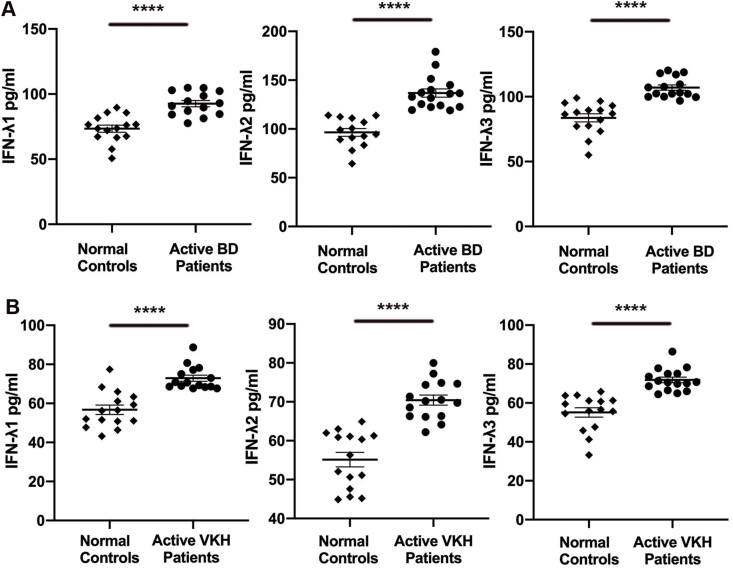
Serum levels of IFN-λs in patients with active BD and active VKH. Serum was collected from active VKH, active BD and normal controls, and the levels of IFN-λs in the serum were measured by ELISA. **(A)** 14–16 active BD patients *vs.* 14–15 normal controls. **(B)** 15–16 active VKH patients *vs.* 15 normal controls. ∗∗∗∗*P* < 0.0001; *P* value was corrected by Bonferroni. BD, Behçet's disease; ELISA, Enzyme-linked immunosorbent assay; VKH, Vogt-Koyanagi-Harada.

To investigate the role of IFN-λs in immune-mediated uveitis, we first examined their expression in patients with active BD and active VKH. PBMCs from active BD and VKH patients showed elevated mRNA expression of IFN-λ1, IFN-λ2, IFN-λ3 and IFNLR1 compared with normal controls (Fig. 1A and B). Serum protein levels of IFN-λ1, IFN-λ2, and IFN-λ3 were also significantly increased in these patients as compared to normal controls (Fig. 2A and B). These results demonstrate that IFN-λ signaling may be involved in the development of these two diseases.

### IFNLR1^−/−^ mice develop exacerbated EAU

**Figure 3:**
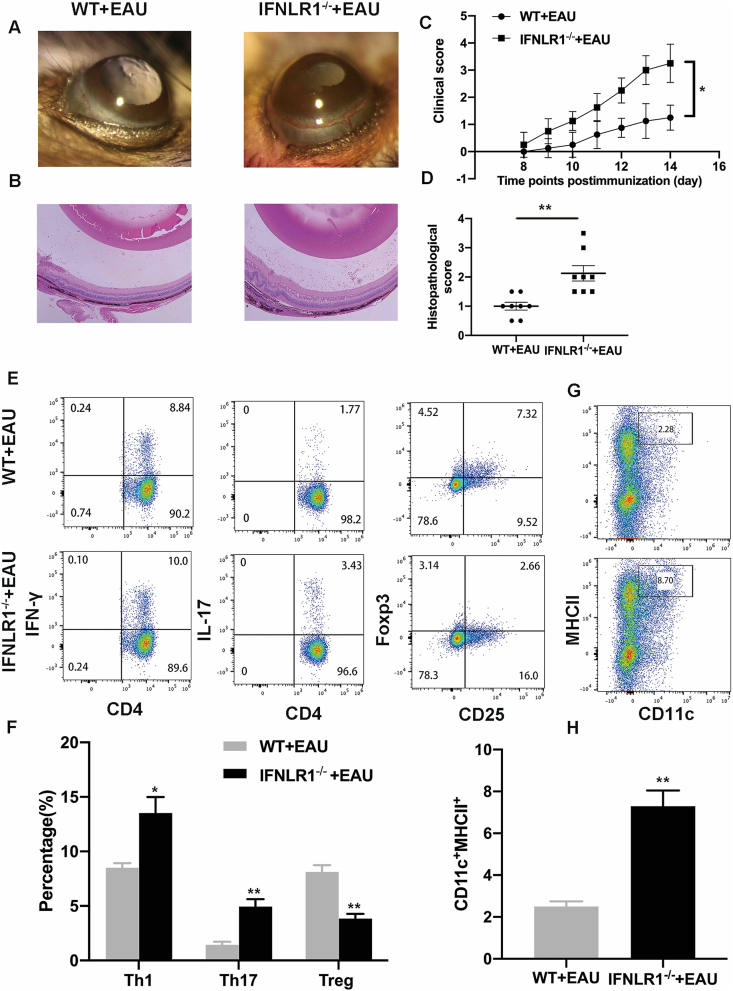
Effect of IFNLR1 deficiency on EAU, and the percentage of Th1, Th17, Treg cells and CD11c^+^MHC class II in splenocytes from IFNLR1^−/−^ EAU mice and WT EAU. **(A, B)** Representative clinical slit-lamp (A) and hematoxylin and eosin (H&E) staining section (B) images of EAU mice on day 14 after immunization. Scale bar: 50 μm. **(C)** Clinical scores were measured from day 8 to day 14 after immunization (the *P* value was evaluated on day 14 after immunization, ∗*P* < 0.05, ∗∗*P* < 0.01). **(D)** Histological scores were assessed by H&E staining on day 14 after immunization. **(E**–**H)** Flow cytometric analysis was performed on splenic cells from the two groups using antibodies against IFN-γ, IL-17, Foxp3, MHC-II and CD11c. (E, G) Representative flow cytometry dot plots. (F, H) Histograms of CD4^+^IFN-γ^+^, CD4^+^IL-17^+^, CD25^+^Foxp3^+^ and CD11c^+^MHCII^+^ (*n* = 4–5 per group). Data are shown as mean ± SEM from two independent experiments. ∗*P* < 0.05, ∗∗*P* < 0.01; *P* value was corrected by Bonferroni. EAU, experimental autoimmune uveitis; WT, wild-type.

To explore the endogenous role of IFN-λ in EAU, WT and IFNLR1^−/−^mice were immunized with human IRBP_651-670_ to induce EAU. Results demonstrated that IFNLR1^−/−^ mice (*n* = 8) developed more severe ocular inflammation compared to WT controls (Fig. 3A–C). Histopathological analysis further revealed that IFNLR1^−/−^ mice exhibited aggravated retinal vasculitis and increased inflammatory cell infiltration relative to WT mice (Fig. 3B–D). To elucidate the mechanisms by which IFNLR1 deficiency exacerbates EAU, splenocytes were isolated on day 14 post-immunization and analyzed for Th1, Th17 and Treg cells. Flow cytometry analysis indicated a significantly elevated frequency of IL-17A^+^CD4^+^ T cells and IFN-γ^+^CD4^+^ T cells, along with a reduced proportion of CD25^+^Foxp3^+^ regulatory T cells in IFNLR1^−/−^ EAU mice compared to WT EAU mice (Fig. 3E and F). Additionally, an increase in the population of splenic CD11c^+^MHCII^+^ antigen-presenting cells was observed in IFNLR1^−/−^ EAU mice (Fig. 3G and H). Collectively, these findings suggest that IFN-λ signaling may help maintain immune homeostasis by modulating the balance between effector Th1/Th17 cells and regulatory T cells, thereby attenuating intraocular inflammation.

### IFNLR1 deficiency induces hyperfunction of DCs in EAU mice

**Figure 4:**
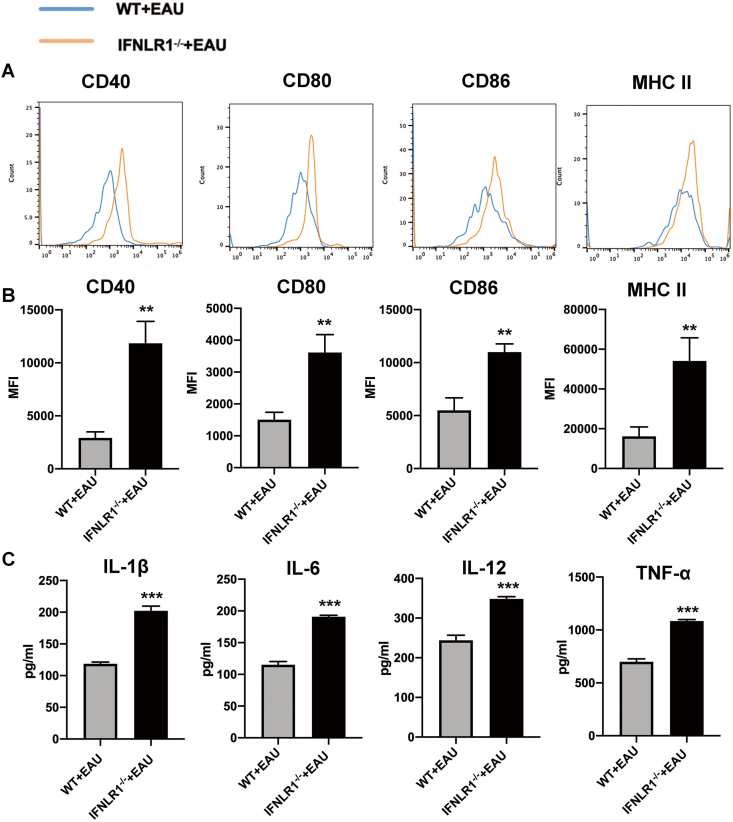
IFNLR1 deficiency regulates the function of DCs. **(A, B)** Flow cytometric analysis of the expression (mean fluorescence intensity [MFI]) of CD40, CD80, CD86, and MHC-II on the splenic DCs from the IFNLR1^−/−^ EAU mice and WT EAU (*n* = 4–5 per group). **(C)** Splenic DCs from the two groups were stimulated with LPS for 24 h, then the supernatants were collected for the detection of IL-1β, IL-6, TNF-α and IL-12 by ELISA (*n* = 8 per group). Data are shown as mean ± SEM from 2 independent experiments. ∗∗*P* < 0.01, ∗∗∗*P* < 0.001; *P* value was corrected by Bonferroni. DCs, dendritic cells; EAU, experimental autoimmune uveitis; ELISA, Enzyme-linked immunosorbent assay; LPS, lipopolysaccharide.

DCs play a critical role in the differentiation of naïve CD4^+^ T cells into distinct T helper subsets through the expression of surface molecules and secretion of cytokines. To further investigate how IFNLR1 deficiency exacerbates EAU, we examined its impact on DCs in the EAU model. Splenic DCs were isolated from WT and IFNLR1^−/−^ EAU mice on day 14 post-immunization and analyzed by flow cytometry for the expression of surface markers. Results revealed a significant upregulation of co-stimulatory molecules, including CD40, CD80, CD86, and MHC II, on splenic DCs from IFNLR1^−/−^ EAU mice compared to those from WT controls (Fig. 4A and B). Furthermore, after stimulation with LPS (100 ng/mL) for 24 h, culture supernatants of splenic DCs from IFNLR1^−/−^ EAU mice exhibited markedly elevated levels of the proinflammatory cytokines IL-1β, IL-6, TNF-α, and IL-12 relative to WT DCs (Fig. 4C). Taken together, these findings suggest that IFN-λ signaling may help maintain immune balance by modulating regulatory DCs, which in turn could suppress Th1 and Th17 responses while promoting regulatory T cell induction, thereby attenuating ocular inflammation.

### IFNLR1 deficiency causes BRB breakdown in EAU mice

**Figure 5:**
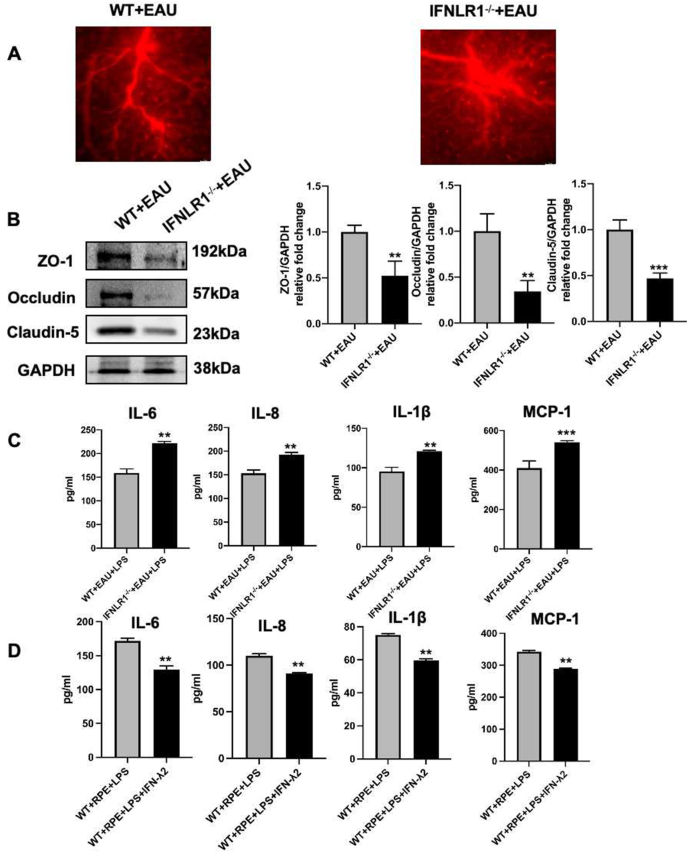
The effect of IFNLR1 deficiency on the function of blood-retinal barrier and RPE cells *in vivo*. **(A)** Representative images of Evans blue staining in retinas from WT EAU mice and IFNLR1^−/−^ EAU mice. Scale bar: 50 μm. **(B)** Representative Western blotting images and quantitative analysis of ZO-1, Claudin-5 and Occludin in retinas from the two groups (*n* = 4 per group). **(C)** Primary RPE cells were harvested from the two groups and cultured with LPS for 24 h, then the supernatants were collected for the detection of IL-6, IL-8, IL-1β, and MCP-1 by ELISA (*n* = 8 per group). **(D)** Primary RPE cells were co-treated with recombinant IFN-λ2 and LPS. The secreted levels of the pro-inflammatory cytokines IL-6, IL-8, IL-1β, and MCP-1 in the supernatants were measured by ELISA (*n* = 5 per group). ∗∗*P* < 0.01, ∗∗∗*P* < 0.001; *P* value was corrected by Bonferroni. EAU, experimental autoimmune uveitis; ELISA, enzyme-linked immunosorbent assay; LPS, lipopolysaccharide; RPE, retinal pigment epithelium; WT, wild-type.

The BRB plays a critical role in the pathogenesis of EAU, with RPE cells being central to retinal homeostasis. BRB disruption is a key event in the development of uveitis. To investigate the impact of IFNLR1 deficiency on RPE cells, we first assessed BRB integrity using Evans blue assay. The results revealed significantly increased leakage of retinal vessels in IFNLR1^−/−^ EAU mice compared with WT EAU controls (Fig. 5A). We further evaluated the effect of IFNLR1 deficiency on the expression of tight junction proteins including zonula occludens-1 (ZO-1), Occludin, and Claudin-5 in RPE cells. Results demonstrated markedly reduced expression of all three proteins in the retinas of IFNLR1^−/−^ EAU mice (Fig. 5B). Additionally, we examined cytokine production by primary RPE cells isolated from the two EAU groups. RPE cells derived from IFNLR1^−/−^ EAU mice secreted significantly higher levels of the proinflammatory factors IL-6, IL-8, IL-1β, and MCP-1 compared to those from WT EAU mice (Fig. 5C). To validate the alterations in inflammatory cytokines induced by exogenous IFN-λ, we administered recombinant IFN-λ2 to mouse RPE cells. The results showed that treatment of RPE cells with recombinant IFN-λ2 markedly suppressed the secretion of these pro-inflammatory factors (Fig. 5D). These findings suggest that IFN-λ signaling might help maintain RPE barrier function by preserving tight junction integrity and suppressing proinflammatory responses, thereby exerting a protective effect against uveitis.

### IFNLR1 deficiency regulates RPE function *in vitro*

**Figure 6:**
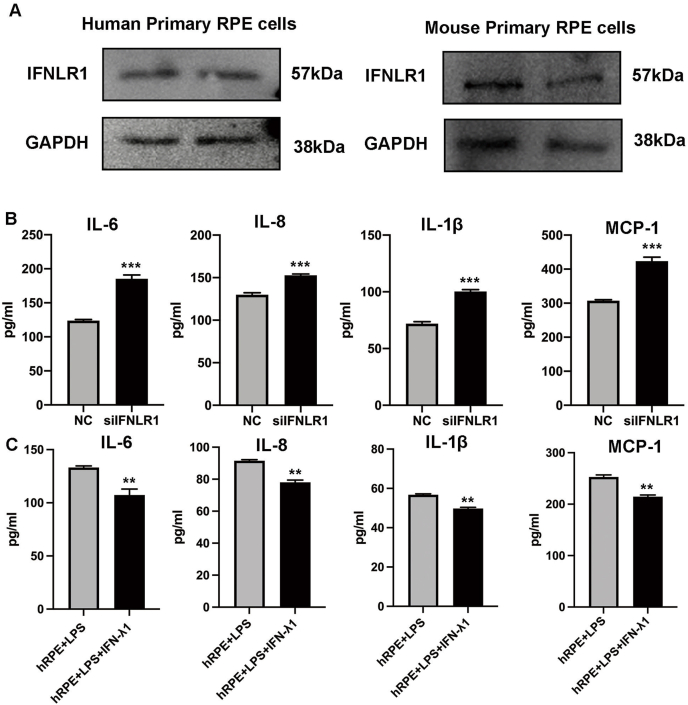
The effect of IFNLR1 deficiency on the function of RPE cells *in vitro*. **(A)** Protein expression of IFNLR1 on the murine and human primary RPE cells was determined by Western blotting. **(B)** Human primary RPE cells were transfected with siIFNLR1 in presence of LPS *in vitro*, then the supernatants were collected for the detection of IL-6, IL-8, IL-1β and MCP-1 by ELISA (*n* = 8/group). **(C)** Human primary RPE cells were cultured with recombinant IFN-λ1 in presence of LPS and the supernatants were collected for the detection of IL-6, IL-8, IL-1β and MCP-1 by ELISA. ∗∗*P* < 0.01, ∗∗∗*P* < 0.001 (*n* = 5–6 per group); *P* value was corrected by Bonferroni. ELISA, enzyme-linked immunosorbent assay; LPS, lipopolysaccharide; RPE, retinal pigment epithelium.

To explore whether IFN-λ signaling can directly regulate RPE function, we first investigated whether IFNLR1 was expressed on mouse and human primary RPE cells. The results showed that both mouse and human primary RPE cells expressed abundant IFNLR1 protein (Fig. 6A). We next transfected human primary RPE cells with siIFNLR1 and treated the cells with recombinant IFN-λ1 to evaluate the impact of IFNLR1 knockdown on the production of proinflammatory cytokines. The results showed that the levels of IL-6, IL-8, IL-1β and MCP-1 were increased in the culture supernatants from siIFNLR1-transfected human RPE cells compared with those without siIFNLR1 (Fig. 6B). In contrast, administration of recombinant IFN-λ1 reduced the secretion of these cytokines (Fig. 6C). Collectively, these data demonstrate that IFN-λ might suppress proinflammatory cytokine release from RPE cells, thereby contributing to the maintenance of epithelial barrier function.

### Identification of DEGs in the RPE cells and DCs from IFNLR1^−/−^ EAU mice by RNA sequencing

To elucidate the molecular mechanisms underlying the exacerbated EAU in IFNLR1^−/−^ mice, we performed mRNA sequencing on RPE cells and DCs isolated from IFNLR1^−/−^ and WT EAU mice. Volcano plots illustrating the DEGs in each cell type are presented in Figure S1. Transcriptomic analysis identified a total of 1160 DEGs in RPE cells, with 961 genes upregulated and 199 downregulated in IFNLR1^−/−^ mice compared to WT controls. In DCs, 2589 DEGs were dysregulated, comprising 1896 upregulated and 693 downregulated genes. Cluster analysis of the DEGs demonstrated a clear segregation of gene expression profiles between IFNLR1^−/−^ and WT EAU mice, with samples within each group clustering together, indicating distinct transcriptomic signatures associated with IFNLR1 deficiency (Fig. S2).

### Identification of the pathways involved in the exacerbation of uveitis in IFNLR1^−/−^ mice

To elucidate the functions and potential biological roles of the DEGs in EAU, we performed GO and KEGG enrichment analyses. Given the heightened autoimmune response observed in IFNLR1^−/−^ mice, we focused specifically on the upregulated DEGs in this model. GO enrichment analysis indicated that T cell activation was the most significantly enriched biological process in both RPE cells and DCs. Other prominently enriched processes in RPE cells included positive regulation of immune response and regulation of leukocyte activation, whereas DCs showed enrichment in lymphocyte differentiation and adaptive immune response.

**Figure 7:**
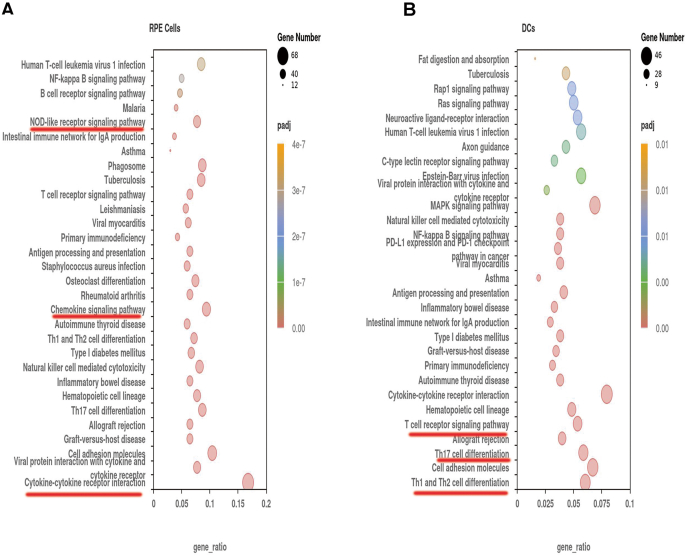
The KEGG enrichment analysis of all differentially expressed genes. The bubble plot shows all significantly upregulated enriched KEGG pathways in retinal pigment epithelium cells **(A)** and dendritic cells **(B)** between IFNLR1^−/−^ EAU and WT EAU group. EAU, experimental autoimmune uveitis; KEGG, Kyoto Encyclopedia of Genes and Genomes; WT, wild-type.

A total of 113 upregulated DEGs in RPE cells and 89 in DCs were associated with T cell activation (Fig. S3). KEGG pathway analysis further revealed that the upregulated DEGs in RPE cells were significantly enriched in immune-related pathways, including cytokine–cytokine receptor interaction, chemokine signaling, and the NOD-like receptor signaling pathway, encompassing NLRP3 and NLRP1 (Fig. 7A). In DCs, the DEGs were primarily involved in T cell-related pathways, such as Th1 and Th2 cell differentiation, T cell receptor signaling pathway and Th17 cell differentiation (Fig. 7B).

### Validation of the role of NLRP3 and NLRP1 in the exacerbation of uveitis in IFNLR1^−/−^ mice

**Figure 8:**
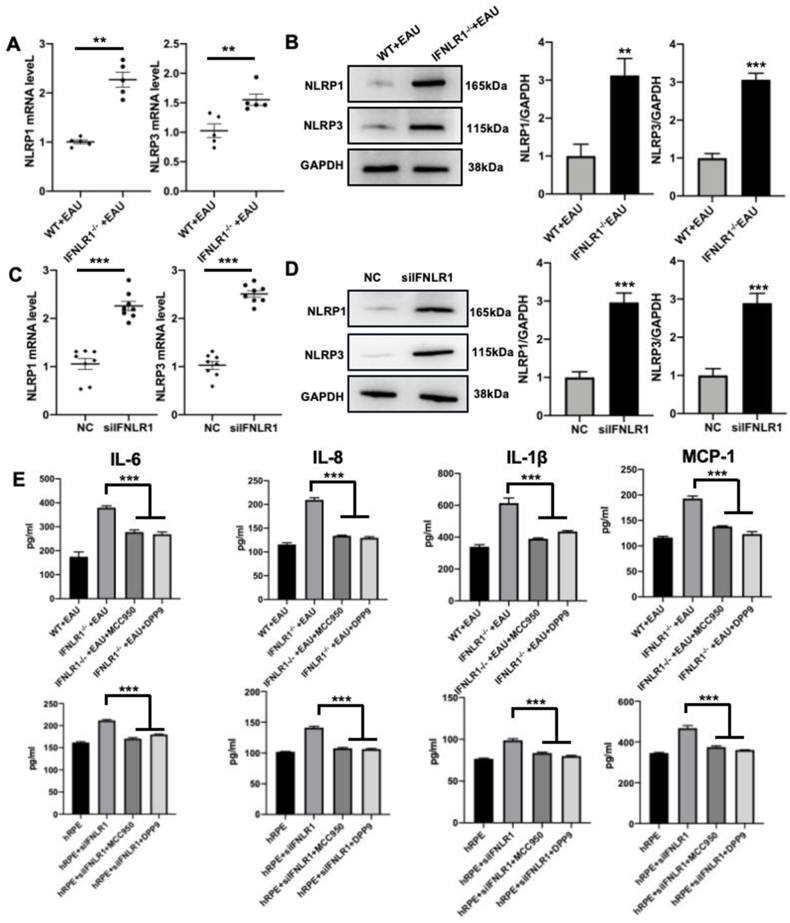
Validation of NLRP3 and NLRP1 in the RPE cells of EAU with IFN-λ deficiency. **(A, B)** The primary RPE cells from IFNLR1^−/−^ EAU mice and WT EAU mice were harvested for the detection of NLRP3 and NLRP1. (A) The mRNA expression of NLRP3 and NLRP1 was detected by RT-qPCR. (B) The representative Western blotting images and histogram (*n* = 4). **(C, D)** Primary hRPE cells were transfected with siIFNLR1, and then the cells were assayed for NLRP1 and NLRP3 expression by RT-qPCR and Western blotting (*n* = 4). **(E)** Primary RPE cells from IFNLR1^−/−^ EAU mice and hRPE cells with siIFNLR1 were treated with or without MCC950 (a potent, selective and small-molecule inhibitor of NLRP3) or DPP9 (a direct inhibitor of NLRP1) in the presence of LPS stimulation, and the supernatants were harvested for the expression of inflammatory cytokines including IL-6, IL-8, MCP-1, IL-1β and TNF-α by ELISA (*n* = 5). ∗∗*P* < 0.01, ∗∗∗*P* < 0.001; *P* value was corrected by Bonferroni. EAU, experimental autoimmune uveitis; ELISA, enzyme-linked immunosorbent assay; LPS, lipopolysaccharide; RPE, retinal pigment epithelium; RT-qPCR, real-time quantitative PCR.

Our KEGG analysis revealed that NLRP1 and NLRP3, key factors known to be involved in uveitis,^37^ were among the DEGs enriched in the NOD-like receptor pathway in RPE cells, prompting further investigation into their role. We therefore designed a study to determine whether NLRs were associated with the exacerbation of uveitis in IFNLR1^−/−^ mice. The results showed upregulated expression of both NLRP1 and NLRP3 at the mRNA and protein levels in the RPE cells of IFNLR1^−/−^ EAU mice as compared to WT EAU mice (Fig. 8A and B). We further performed *in vitro* experiment to confirm their involvement in the exacerbation of EAU. The results revealed a significantly increased expression of NLRP1 and NLRP3 in human primary RPE cells transfected with siIFNLR1 compared to those without siIFNLR1 (Fig. 8C and D). These results revealed a novel signaling pathway of IFN-λ in alleviating the pathogenesis of EAU by inhibiting the expression of NLRP1 and NLRP3 in RPE cells.

### Inhibition of NLRP1 and NLRP3 reduces the expression of proinflammatory cytokines by RPE cells

Based on the observed upregulation of NLRP1 and NLRP3 in RPE cells from IFNLR1^−/−^ EAU mice and in IFNLR1-silenced human primary RPE cells, we next investigated whether inhibiting NLRP1 and NLRP3 could attenuate proinflammatory cytokine secretion in these cells. RPE cells isolated from IFNLR1^−/−^ EAU mice and human RPE cells transfected with siIFNLR1 were treated with MCC950 (a specific small-molecule inhibitor of NLRP3) or DPP9 (a direct NLRP1 inhibitor).^38^^,^^39^ The secretion of inflammatory cytokines, including IL-6, IL-8, MCP-1, IL-1β, and TNF-α, was measured by ELISA. As shown in Figure 8E, both MCC950 and DPP9 treatment significantly reduced the release of these cytokines in IFNLR1-deficient RPE cells from both human and mouse. To investigate whether IFN-λ regulates RPE barrier function through NLRP1 and NLRP3 expression, the expression levels of tight junction proteins ZO-1, Claudin-5, and Occludin were assessed *in vitro* using ELISA. The results showed that the levels of these tight junction proteins were upregulated in IFNLR1-knockdown RPE cells following treatment with the NLRP3 inhibitor MCC950 or the NLRP1 inhibitor DPP9 in the presence of LPS, compared to siIFNLR1-transfected RPE cells without intervention (Fig. S4). Subsequently, apoptosis was evaluated in hRPE cells with IFNLR1 knockdown after treatment with NLRP1 or NLRP3. However, no significant differences in apoptosis were observed among the groups treated with NLRP1 or NLRP3 (Fig. S5). These findings indicate that IFN-λ signaling exerts a protective effect by downregulating the expression of NLRP1 and NLRP3. This regulation inhibits the production of pro-inflammatory cytokines by RPE cells, contributes to the preservation of tight junction proteins, including ZO-1, Claudin-5 and Occludin, and ultimately mitigates the severity of uveitis.

## Discussion

Studies have shown that IFN-λs play different roles in a various diseases. The present study demonstrated their protective effect on EAU in view of the following aspects. First, we demonstrated a significantly increased expression of IFN-λs and IFNLR1 in patients with active VKH and active BD. Second, IFNLR1^−/−^ mice developed aggravated EAU in association with over-activation of Th1 and Th17 cell response and a downregulated Treg cells *in vivo*. Third, splenic DCs from IFNLR1^−/−^ EAU mice expressed increased levels of MHC II and positive costimulatory molecules, and secreted higher levels of pro-inflammatory cytokines (IL-1β, IL-6, TNF-α and IL-12). Fourth, IFNLR1 deficiency led to disruption of the tight junctions in the RPE and increased secretion of pro-inflammatory cytokines. Finally, RNA sequencing and bioinformatic analyses revealed that NLRP1 and NLRP3 were both upregulated in the RPE cells from IFNLR1^−/−^ EAU mice. IFNLR1 deficiency resulted in elevated expression of NLRP1 and NLRP3 in RPE cells *in vitro*. Notably, knockdown of NLRP1 and NLRP3 attenuated the expression of pro-inflammatory cytokines and upregulate tight junction proteins in hRPE cells transfected with siIFNLR1. Collectively, our study indicated a protective role of IFN-λ signaling in autoimmune uveitis.

IFN-α, a member of Type I interferon, has been applied in the clinical treatment for certain types of uveitis, such as BD, idiopathic panuveitis, and pediatric uveitis.^40^ It has relatively more side effects due to its widespread expression of receptors, such as high fever, fatigue, headache, myalgia, joint pain, nausea and loss of appetite. In contrast, IFN-λ has fewer side effects, because its receptor is restrictively expressed on specific cells and barrier tissues.^41^ It is reported that the combination of corticosteroids and adalimumab is superior to corticosteroids plus ciclosporin in reducing uveitis relapse rates,^42^ with IFN-α-2a plus corticosteroids potentially offering intermediate efficacy.^43^ The findings of this study suggest that IFN-λ could serve as a promising alternative therapeutic agent. Similar to the aforementioned treatments,^44^ it effectively controls inflammation; moreover, it exerts more precise immunomodulatory effects at ocular barrier sites, potently inhibits the secretion of proinflammatory factors, and may thereby rapidly attenuate disease progression.

To address whether IFN-λ plays a protective role in uveitis, we examined its expression in patients with uveitis and its role in EAU. The results showed elevated mRNA and protein expression of IFN-λ1, IFN-λ2, IFN-λ3 and IFNLR1 in PBMCs and serum from patients with active BD and active VKH patients. The results suggest that the up-regulation of IFN-λ and its receptor may be a common feature in the pathogenesis of those two types of uveitis. Our results are in line with a previous report showing the increased concentration of IFN-λ in blood and affected tissues in several autoimmune diseases, including rheumatoid arthritis (RA), primary sjögren syndrome and systemic sclerosis (SSc).^26^ These results suggest that IFN-λ signaling is involved in the pathogenesis of these diseases; however, it may play different roles in different diseases.

To further investigate the role of IFN-λs *in vivo*, we used the IFNLR1^−/−^ mice to induce EAU and found that the IFNLR1 deficiency could lead to more severe EAU, revealing its protective role in autoimmune uveitis. Our findings are consistent with previous reports demonstrating the immunoregulatory effect of IFN-λs in several autoimmune mouse models, such as experimental autoimmune encephalomyelitis (EAE), dextran sulfate sodium (DSS)-induced colitis, and graft-versus-host disease (GVHD).^45^, ^46^, ^47^ However, IFN-λs have been reported to play a pro-inflammatory role in a mouse model of SLE.^48^ Although the mechanisms underlying the discrepancy in the role of IFN-λs in various disease models are not well understood, these data indicate that IFN-λs may exert complex immunoregulatory functions and contribute different effects under varying pathophysiological conditions.

As overactivation of Th1/Th17 cells and the reduction of frequency and function of Treg cells are involved in initiating and progressing of uveitis, we further investigate whether IFN-λs exert their role by regulating the imbalance of pathogenic Th1/Th17 cells and Treg cells. *In vivo* experiments with IFNLR1^−/−^ mice showed that IFNLR1 deficiency was associated with a significantly increased frequency of Th1 and Th17 cells and a decreased frequency of Treg cell population in EAU mice. These results suggest that IFN-λ signaling may play a protective role by regulating the homeostasis of pathogenic Th17 and Th1 cells and regulatory T cells. Our results are consistent with a previous report showing that IFN-λ signaling in macrophages inhibited encephalitogenic Th17 cell expansion *in vitro* in EAE.^49^ However, they failed to find an inhibitory effect of IFN-λ signaling on the Th1 response. This discrepancy may be explained by IFN-λ exerting its therapeutic effects through different mechanisms in different models.

DCs have been shown to play a critical role in the differentiation of Th17/Th1 and Treg cells.^50^ They can induce Treg cell differentiation by secreting IL-10 and TGF-β, and promote Th1 and Th17 cell differentiation by secreting IL-6, IL-1β, IL-12 and TNF-α, and by upregulating costimulatory molecules.^51^ In this study, we investigated the effect of IFN-λ signaling on DCs and found that DCs from IFNLR1^−/−^ EAU mice exhibited increased expression of costimulatory molecules, including CD40, CD80, CD86 and MHC II, and secreted higher levels of the pro-inflammatory cytokines IL-6, IL-1β, IL-12 and TNF-α.

These results suggest that IFN-λ signaling induces a regulatory phenotype in mouse splenic DCs, which could promote the expansion of Foxp3^+^Treg cells and inhibit Th1 and Th17 polarization. Taken together, IFN-λ may exert its inhibitory effect on Th1 and Th17 cells and its induction effects on Treg cells via modulating DC function. Moreover, it is reported that IFN-λ stimulation in plasmacytoid pDCs triggers phosphorylation of STAT1, STAT3, and STAT5, along with induction of IRF-3 and IRF-7,^52^ which are key transcription factors for IL-28A/IL-28B gene expression. In contrast, STAT6 phosphorylation is not observed.^53^ However, the mechanisms governing this differential activation of STAT and IRF signaling pathways by IFN-λ require further elucidation.

It has been shown that BRB breakdown and the homing of peripheral pathogenic Th1 and Th17 cells to the eye are critical to the development of uveitis.^54^ The integrity of the BRB is attributed predominantly to tight junctions. In this study, we found IFN-λ signaling deficiency resulted in an increased vascular leakage and deterioration of the BRB integrity. The RPE cells from IFNLR1^−/−^ EAU mice secreted higher levels of IL-6, IL-8, IL-1β, and MCP-1. We validated this result in human primary RPE cells, and the same trend of upregulation of proinflmmatory factors was observed when IFNLR1 was knocked down. Our findings are consistent with those reported previously in EAE, whereby IFNLR1^−/−^ mice displayed increased blood–brain barrier (BBB) permeability after infection of West Nile virus, and the administration of IFN-λ2 enhances BBB integrity *in vivo*.^49^ However, IFN-λ signaling was reported to disrupt the epithelial barrier function in the lung. The reasons for these conflicting effects at the barrier sites are not fully understood. One possible explanation may be the higher expression of IFNLR1 on pulmonary epithelial cells, which is able to induce sufficient STAT1 activation and therefore activate the IRF1 proinflammatory gene program.^55^ Further research is needed to understand the specific role of IFN-λ in different epithelial cells and the mechanisms involved. Taken together, these findings indicate that IFN-λ signaling exerts a protective role in both mouse and human RPE cells and preserves BRB integrity in EAU.

To further elucidate the mechanisms by which IFN-λ signaling influences RPE function, we conducted RNA sequencing on RPE cells. Transcriptomic analysis revealed that the DEGs were predominantly enriched in the NOD-like receptor signaling pathway. Specifically, the expression of NLRP1 and NLRP3 was significantly upregulated in RPE cells derived from IFNLR1^−/−^ EAU mice, as well as in IFNLR1-silenced RPE cells. Importantly, blockade of NLRP1 and NLRP3 signaling attenuated the secretion of proinflammatory cytokines from RPE cells and alleviated the breakdown of tight junction proteins (The expression of tight junction proteins was analyzed by ELISA^56^; subsequent analyses using RT-qPCR and Western blot are planned to further evaluate this expression). These data demonstrate that IFN-λ signaling suppresses the expression of NLRP1 and NLRP3 in uveitis, and that both molecules contribute critically to the hyperinflammatory phenotype observed in IFNLR1-deficient RPE cells under EAU conditions. In support of this, previous studies have documented overactivation of the NLRP3 inflammasome in uveitis patients, and pharmacological inhibition of NLRP3 has been shown to alleviate EAU in mouse models.^57^^,^^58^ Collectively, these findings suggest that the protective effect of IFN-λ on RPE cells may be mediated by the inhibition of the NLRP3/NLRP1 signaling pathway. Beyond its role in RPE cells, IFN-λs also mediate protective functions through NLR signaling at other barrier sites. Specifically, IFN-λ signaling acts as a negative regulator of Japanese encephalitis virus (JEV) infection in brain microvascular endothelial cells (BMECs).^59^ Furthermore, IFN-λ1 treatment enhances BBB integrity in human BMECs by mitigating NLRP3 inflammasome, highlighting its role in maintaining barrier stability and antiviral defense.^60^ Ahn et al^61^ reported that IFN-λ regulates the airway epithelial barrier to promote immune cell trafficking to sites of infection. This effect was evidenced in a murine model of *Klebsiella pneumoniae* ST258 (KP35) pneumonia, where IFNLR1^−/−^ mice exhibited protection against bacteremia. Mechanistically, IFN-λ signaling reduces epithelial barrier integrity, thereby facilitating immune cell recruitment but concurrently increasing susceptibility to *Klebsiella pneumoniae* invasion. Collectively, depending on the pathological context, IFN-λ can manifest dual functionality at barrier sites, exerting either protective or detrimental effects in various inflammatory diseases.

Although our experiments demonstrated a protective role of IFN-λs in RPE cells, this study has several limitations. We did not analyze the effects of different IFN-λ subtypes on RPE cells. Different IFN-λ subtypes could potentially have different immunoregulatory functions, as variations in their affinity to IFNLR1, stability and tissue distribution may lead to functional differences. The study cohort was limited to 11 to 16 participants per group. Although elevated serum IFN-λ levels were observed in the patient group, the statistical efficacy and sufficient persuasiveness were constrained by the limited sample size.

In summary, this study reveals that IFN-λs may exert a protective effect against EAU by suppressing autoreactive Th1 and Th17 cell responses and promoting the expansion of Tregs. Furthermore, IFN-λs appear to help maintain immune homeostasis by modulating regulatory DCs, which in turn attenuate Th1 and Th17 activity while enhancing Treg induction, collectively leading to the alleviation of ocular inflammation. Our findings also suggest that IFN-λ signaling contributes to the preservation of the BRB and restrains pro-inflammatory cytokine secretion from RPE cells, likely via regulation of the NLRP1/NLRP3 signaling pathway. These results imply that IFN-λs may serve as a promising therapeutic target in uveitis. Further mechanistic studies on IFN-λ signaling are needed and could provide new insights into the prevention and treatment of autoimmune diseases.

## CRediT authorship contribution statement

**Wujiao Wang:** Writing – original draft. **Qingfeng Wang:** Visualization. **Jinyu Cai:** Investigation. **Guannan Su:** Conceptualization. **Wanyun Zhang:** Data curation. **Shuai Su:** Data curation. **Yunfan Zheng:** Methodology. **Chaokui Wang:** Supervision. **Peizeng Yang:** Supervision, Conceptualization.

## Data availability

Data in this manuscript will be made available on request.

## Funding

This study is supported by the National Natural Science Foundation Key Program (China) (No. 82230032), the National Natural Science Foundation Project (No. 82371046), Chongqing Natural Science Foundation Project (China) (No. CSTB2023NSCQ-MSX0306), Chongqing Science and Technology Bureau Mountaineering Project (China) (No.cyyy-xkdfjhjcyj-202301,cyyy-xkdfjh-lcyj-202303, cyyy-xkdfjhcgzh202302), the Science and Technology Research Program of Chongqing Municipal Education Commission (China) (No. KJZD-K202300405) and Chongqing graduate research innovation project (China) (No. CYS21220).

## Conflict of interests

No conflicting relationship exists for any author.
