# Supplementary material for: Type III interferons attenuates Th1/Th17 cell pathogenicity and regulates retinal pigment epithelium cells via NLRP1/NLRP3 signaling axis in autoimmune uveitis
Source: Genes Dis. 2025 Nov 27;13(5):101957. doi: 10.1016/j.gendis.2025.101957 (PMC13276144; doi:10.1016/j.gendis.2025.101957)
Supplement: Multimedia component 1 [file mmc1.docx]

**Supplementary figure 1**


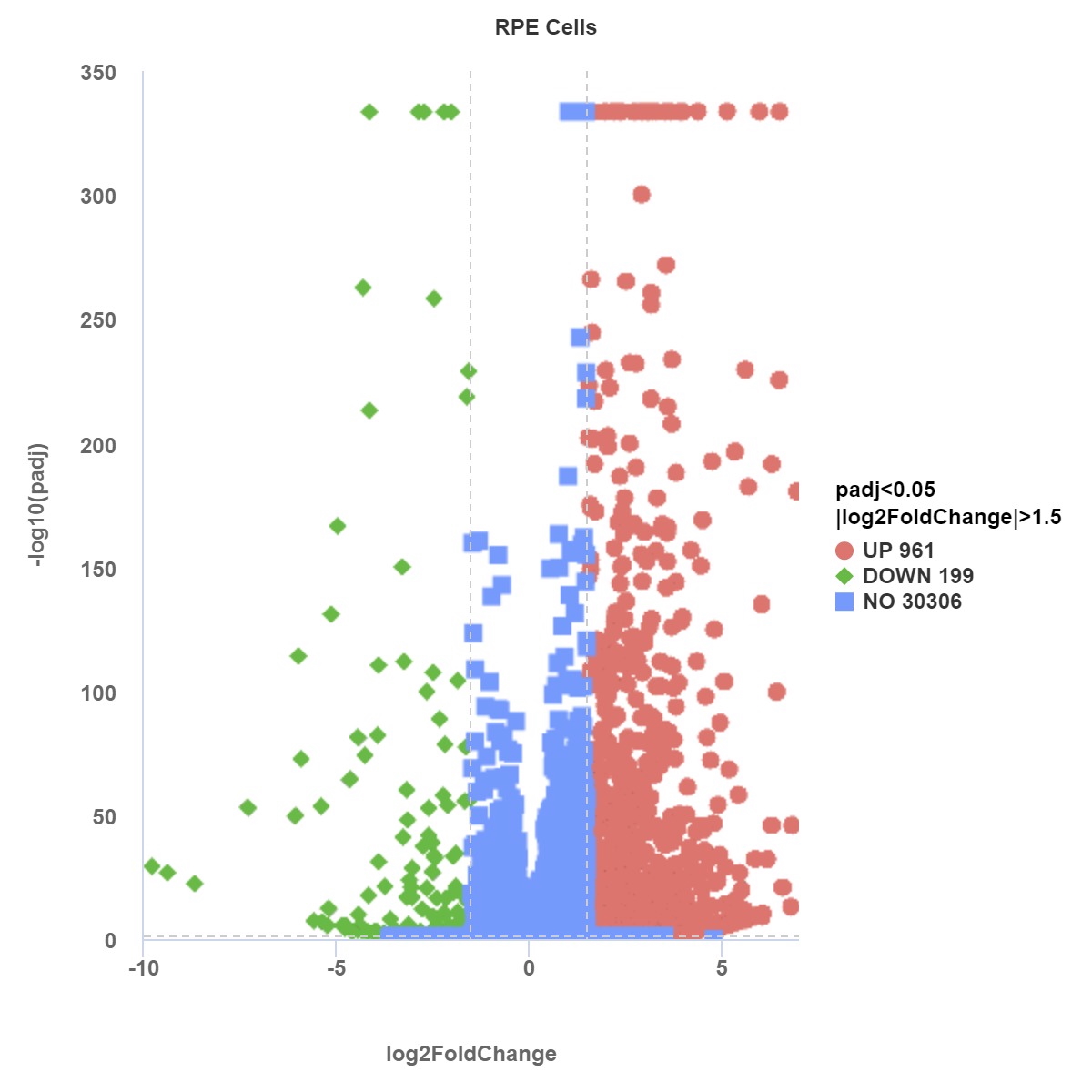


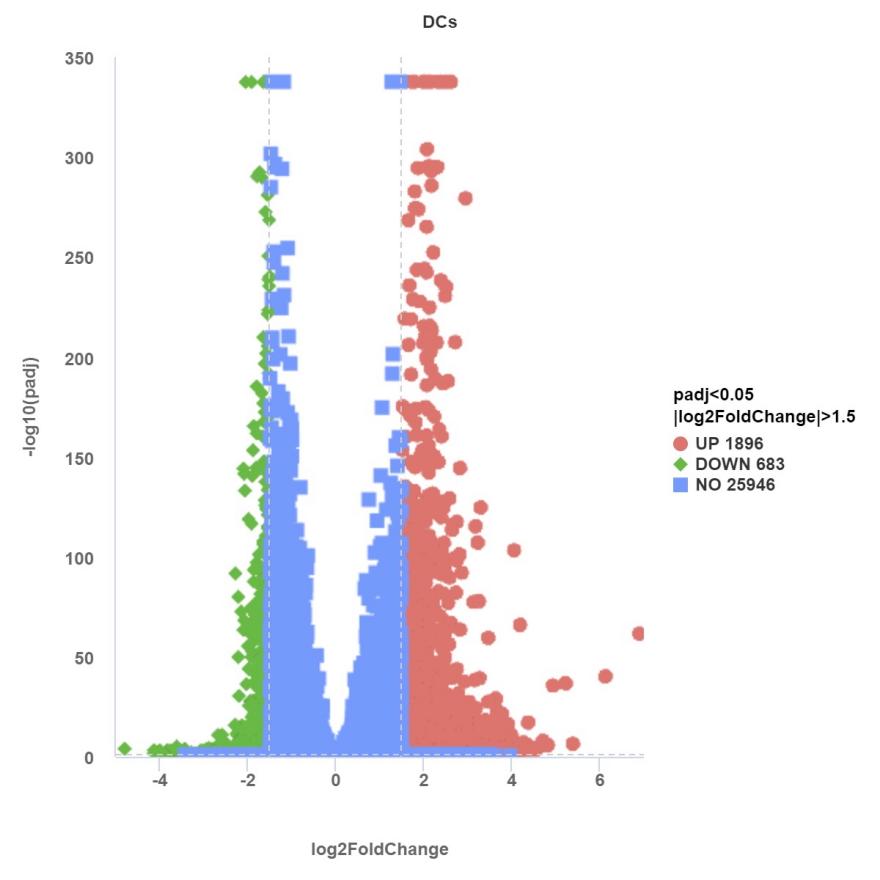


**Legend.** Volcano plots showed DEGs in the RPE cells and DCs between the IFNLR1^-/-^ EAU group (knockout (KO) n = 6) and the WT EAU group ( n = 6). The red dots represent upregulated DEGs, whereas the green dots represent the downregulated DEGs. Nonchanged genes are shown in blue color.

**Supplementary figure 2**

**DCs**


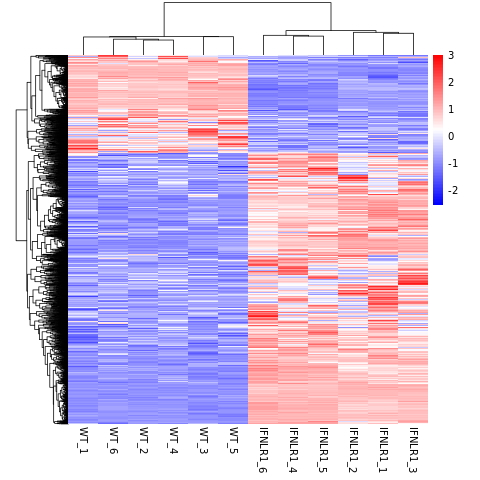


**RPE**


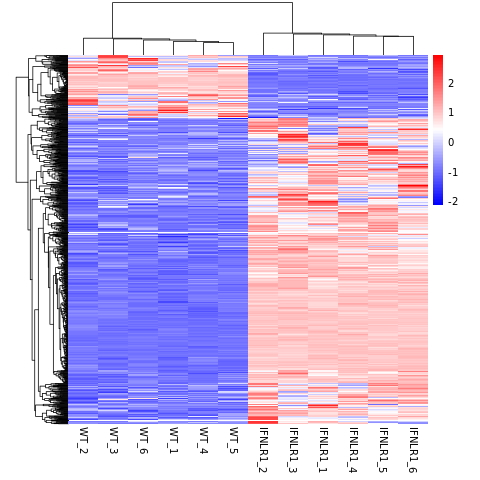


**Legend.** The heat map of the DEGs between these two groups in RPE cells and DCs. DEGs, differentially expressed genes; IFNLR1, the RPE cells or DCs sample from the IFNLR1^-/-^ EAU group; WT, the the RPE cells or DCs sample from WT EAU groups.

**Supplementary figure 3**

**DCs**

**
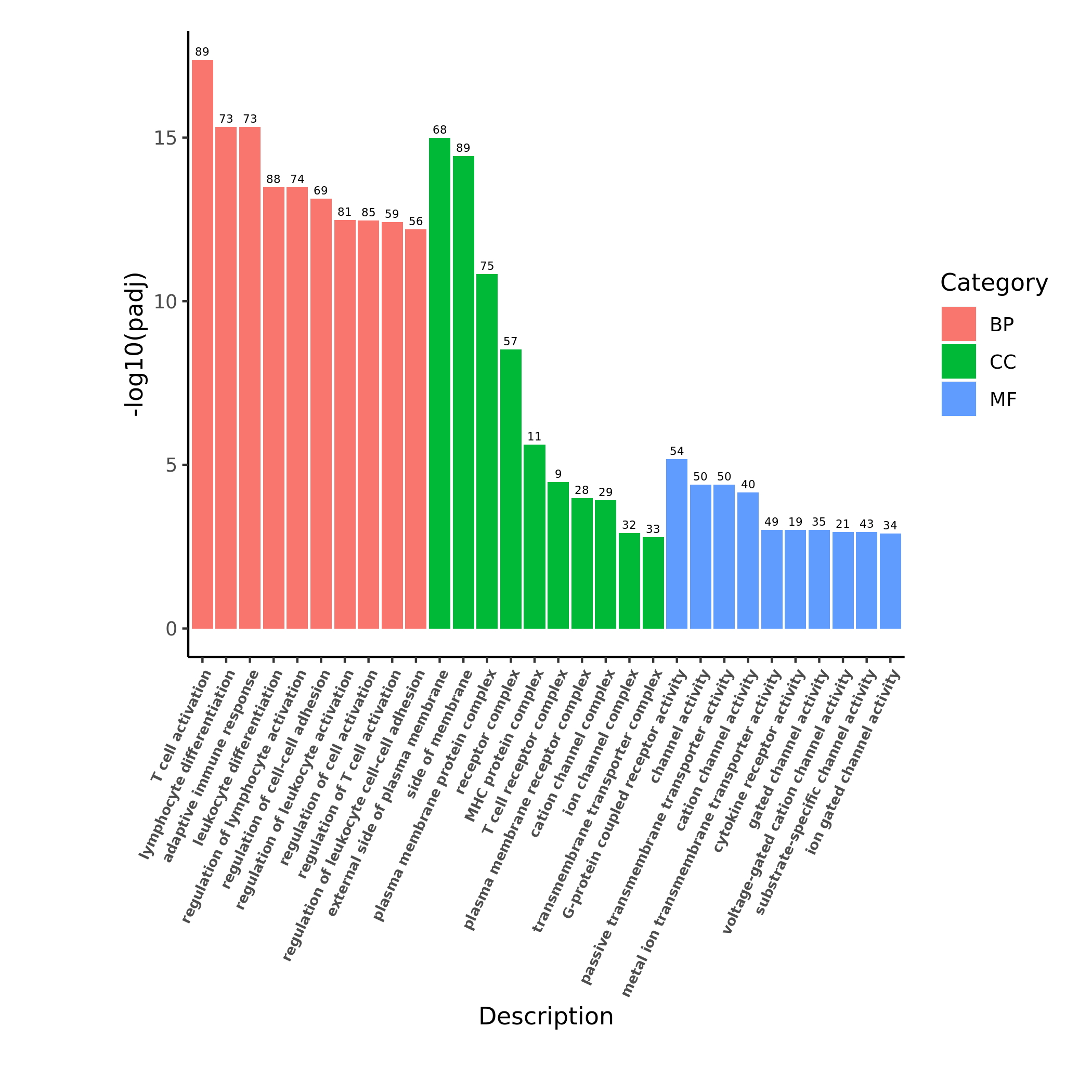
**

**RPE**

**
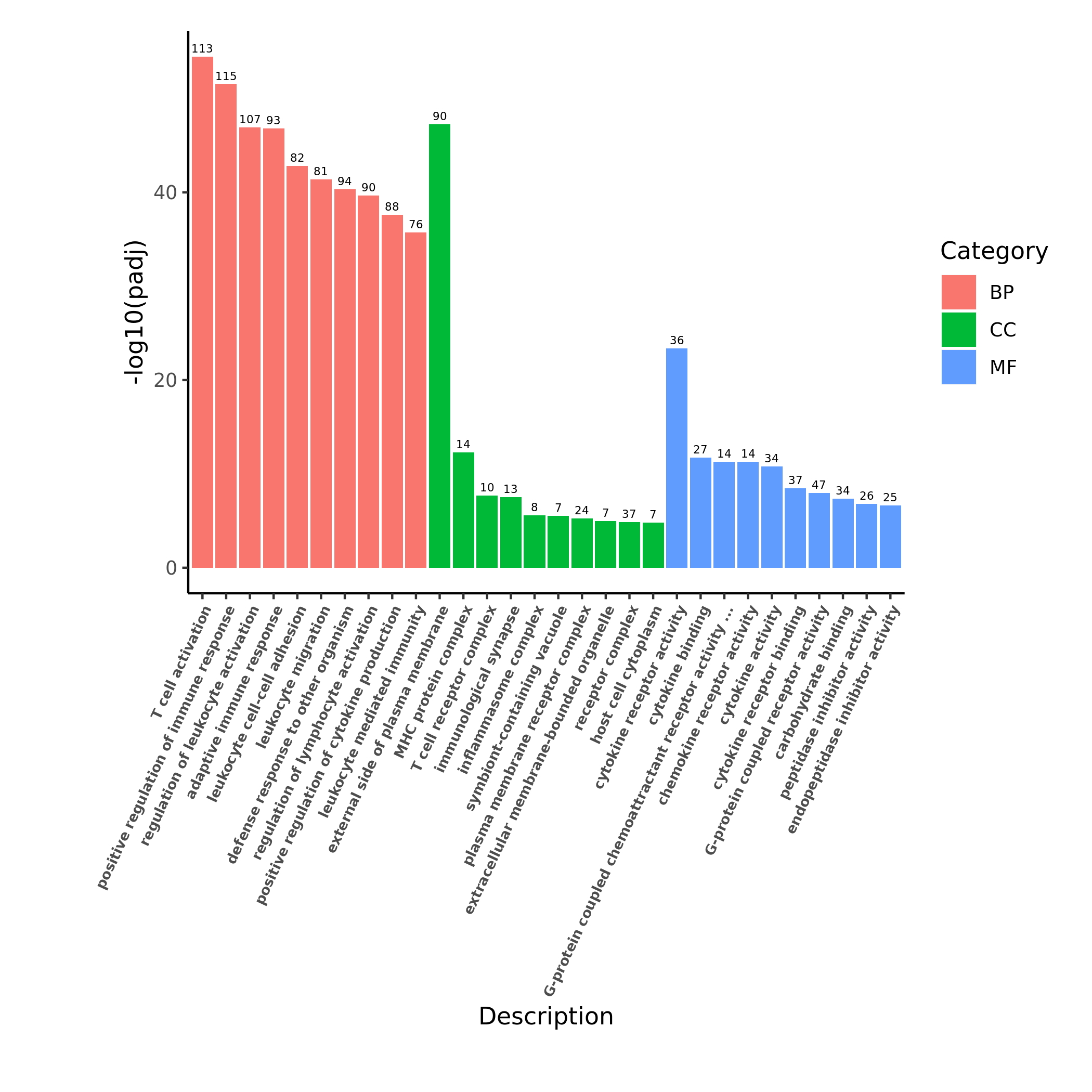
**

**Legend.** The GO enrichment analysis of all DEGs of DCs and RPE cells. The bar graph shows the ten most significantly enriched GO items in each category, including biological process (BP), cellular component (CC), and molecular function (MF).

**Supplementary figure 4**

**Claudin-5**

***

***

****

**Occludin**

**ZO-1**


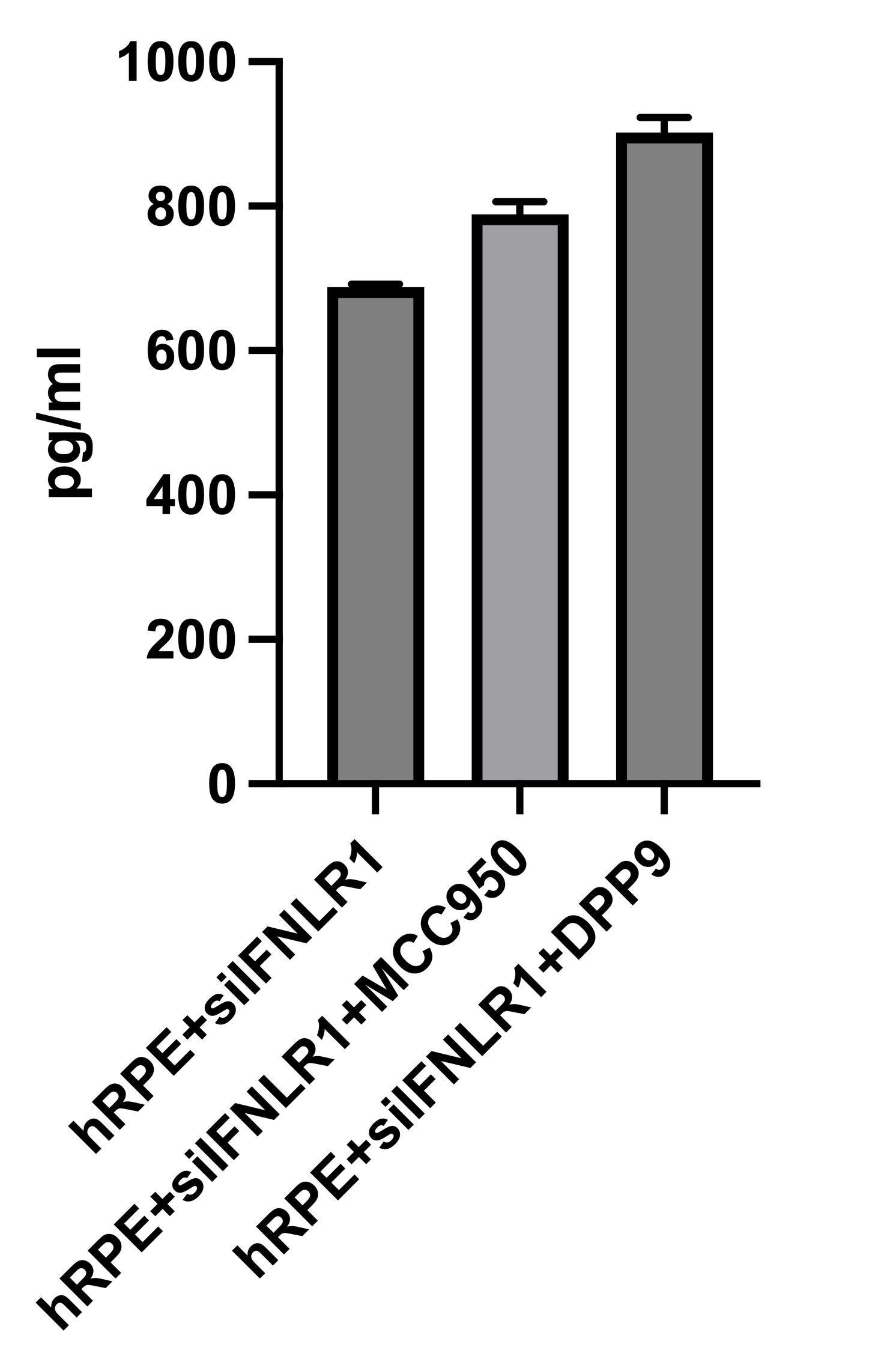
**
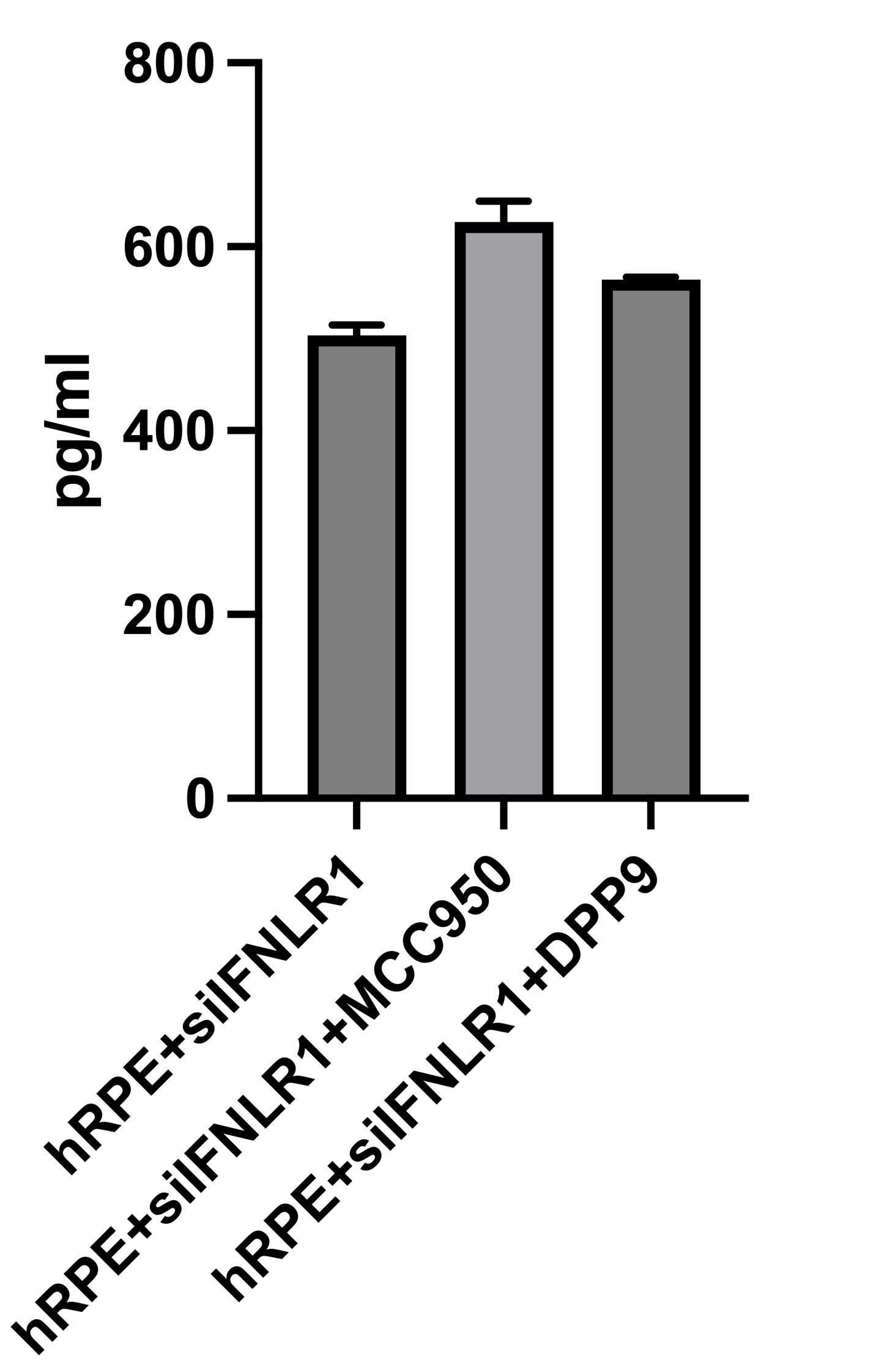

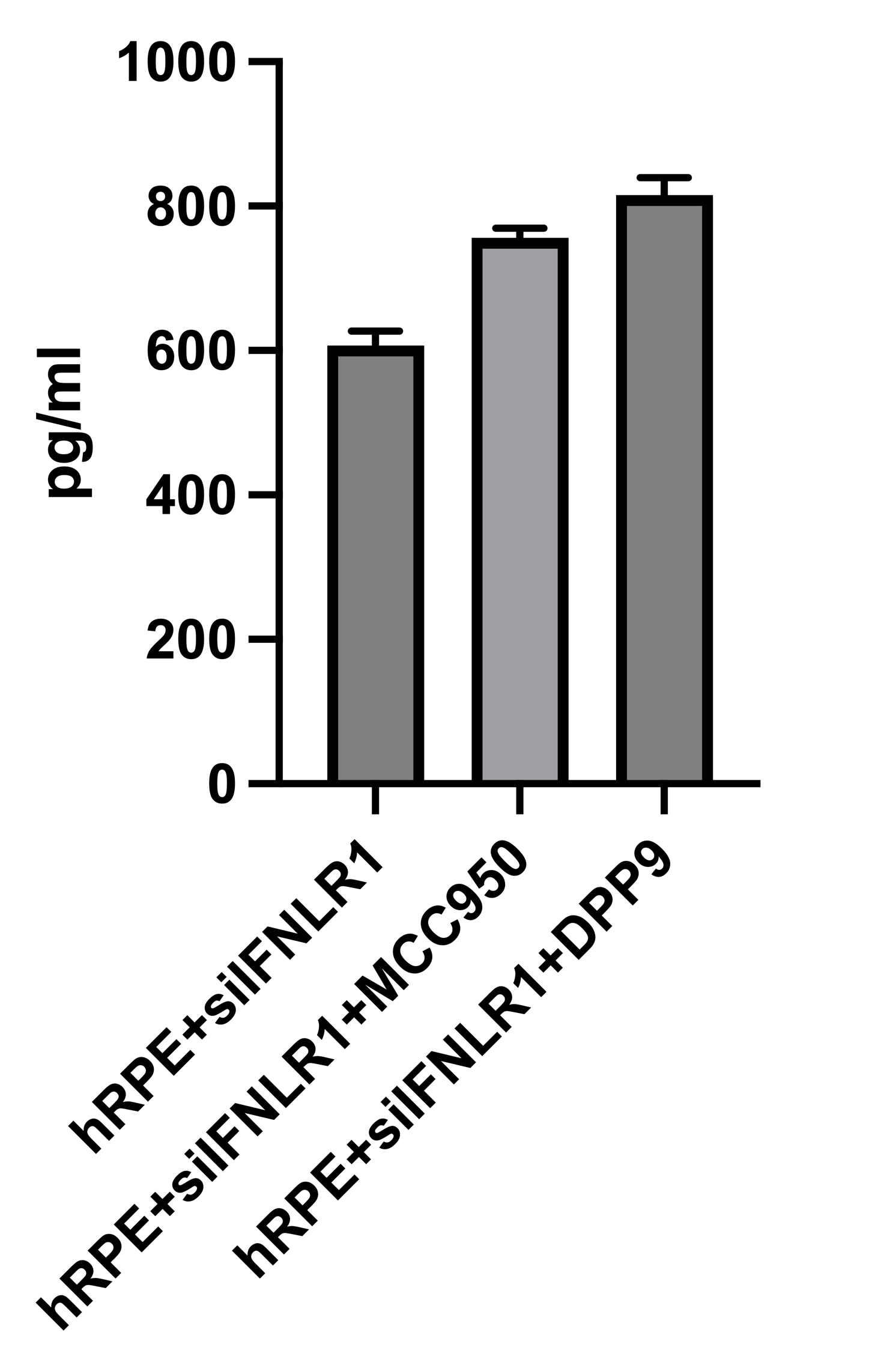
**

**

**

*

**Legend.** The siIFNLR1 RPE cells were treatmented with the NLRP3 inhibitor MCC950 or the NLRP1 inhibitor DPP9 in the presence of LPS. The expression levels of tight junction proteins ZO-1, Claudin-5, and Occludin in hRPE+siIFNLR1, hRPE+siIFNLR1+MCC950 as well as hRPE+siIFNLR1+DPP9 were assessed in vitro using ELISA. *p < 0.05, **p < 0.01, ***p < 0.001, ****p < 0.0001

**Supplementary figure 5**


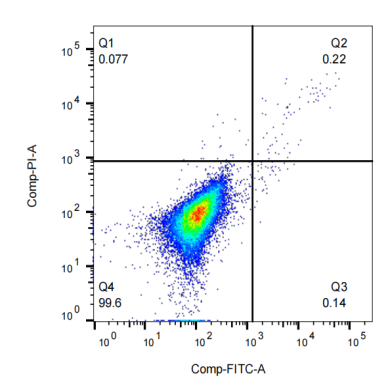

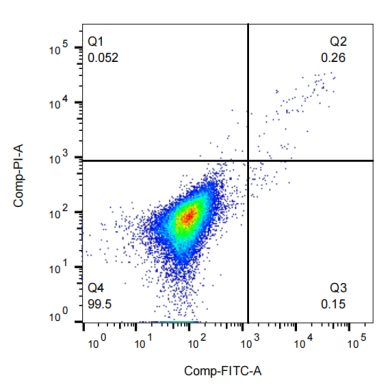

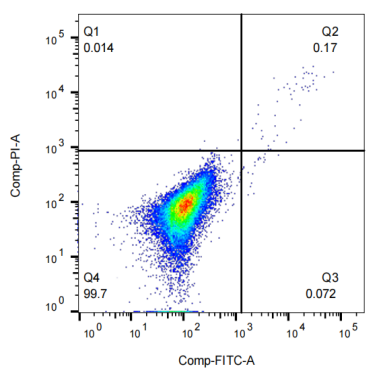


A

**RPE+LPS+siIFNLR1 RPE+LPS+siIFNLR1+NLRP3 RPE+LPS+siIFNLR1+NLRP1**


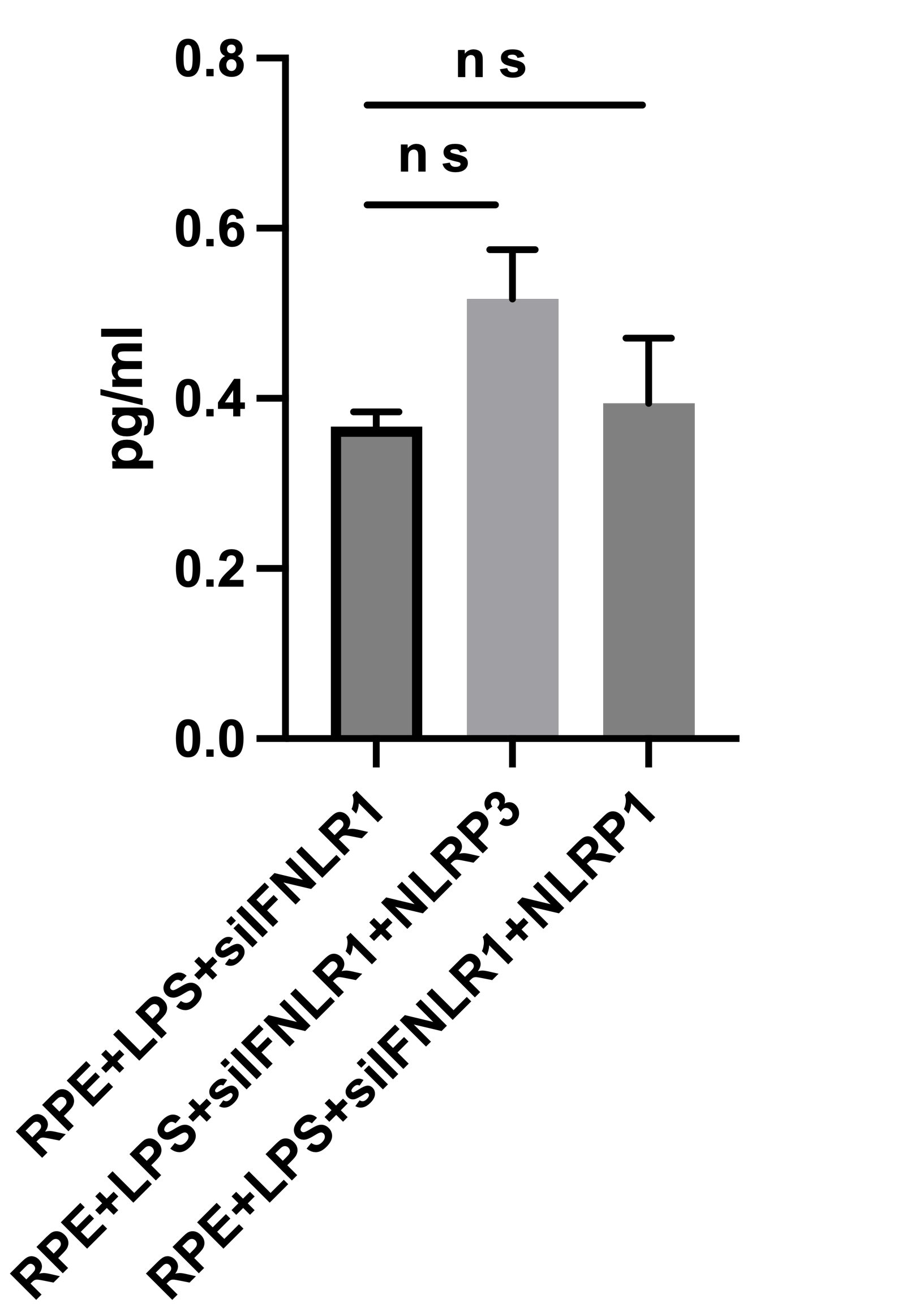


B

**Legend.** Human RPE cells were primed with LPS (100 ng/mL for 12 hours) and pre-incubated in DMEM/F12 media for 1 hour. The cells with siIFNLR1 were subsequently treated with NLRP3 agonists (nigericin at 6 μM for 2 hours; monosodium urate (MSU) at 250 μg/mL for 6 hours; or silica at 250 μg/mL for 6 hours) or an NLRP1 agonist (L18-MDP at 100 μg/mL for 16 hours) to activate the inflammasomes, and the cells were harvested for apoptosis analysis by staining. (A) Representative flow cytometry dot plots. (B) Histograms of RPE+LPS+siIFNLR1, RPE+LPS+siIFNLR1+NLRP3 and RPE+LPS+siIFNLR1+NLRP1. ns means no significance.
